# Sphingomyelin Synthases Regulate Protein Trafficking and Secretion

**DOI:** 10.1371/journal.pone.0023644

**Published:** 2011-09-27

**Authors:** Marimuthu Subathra, Asfia Qureshi, Chiara Luberto

**Affiliations:** Department of Biochemistry and Molecular Biology, Medical University of South Carolina, Charleston, South Carolina, United States of America; Thomas Jefferson University, United States of America

## Abstract

Sphingomyelin synthases (SMS1 and 2) represent a class of enzymes that transfer a phosphocholine moiety from phosphatidylcholine onto ceramide thus producing sphingomyelin and diacylglycerol (DAG). SMS1 localizes at the Golgi while SMS2 localizes both at the Golgi and the plasma membrane. Previous studies from our laboratory showed that modulation of SMS1 and, to a lesser extent, of SMS2 affected the formation of DAG at the Golgi apparatus. As a consequence, down-regulation of SMS1 and SMS2 reduced the localization of the DAG-binding protein, protein kinase D (PKD), to the Golgi. Since PKD recruitment to the Golgi has been implicated in cellular secretion through the trans golgi network (TGN), the effect of down-regulation of SMSs on TGN-to-plasma membrane trafficking was studied. Down regulation of either *SMS1* or *SMS2* significantly retarded trafficking of the reporter protein vesicular stomatitis virus G protein tagged with GFP (VSVG-GFP) from the TGN to the cell surface. Inhibition of SMSs also induced tubular protrusions from the trans Golgi network reminiscent of inhibited TGN membrane fission. Since a recent study demonstrated the requirement of PKD activity for insulin secretion in beta cells, we tested the function of SMS in this model. Inhibition of SMS significantly reduced insulin secretion in rat INS-1 cells. Taken together these results provide the first direct evidence that both enzymes (SMS1 and 2) are capable of regulating TGN-mediated protein trafficking and secretion, functions that are compatible with PKD being a down-stream target for SMSs in the Golgi.

## Introduction

Sphingomyelin synthase (SMS), also known as phosphatidylcholine∶ceramide cholinephosphotransferase, is the last enzyme in the sphingomyelin (SM) synthetic pathway. It synthesizes SM by transferring the phosphorylcholine moiety from phosphatidylcholine (PC) onto ceramide, thus producing not only SM but also diacylglycerol (DAG) [1], [2]. The mammalian SMS family is composed of two members (namely SMS1 and SMS2) that use the same reaction chemistry and are encoded by two distinct genes [3], [4]. Thus far, only few features that differentiate SMS1 and SMS2 have been identified. First, the specific ability of SMS2 to use phosphatidylethanolamine (PE) as head group donor in addition to PC [5]; second, the presence of a sterile alpha motif at the N-terminus of SMS1 (absent in SMS2), whose function remains unknown [6]; and third, their subcellular localization [3], [6], [7], [8]. In fact, SMS1 is localized at the Golgi apparatus whereas SMS2 is localized at the Golgi and plasma membrane by virtue of S-palmitoylation at the COOH terminus [7].

Given the biochemical reaction modulated by SMSs, three features have been considered as potential platforms for a critical role of these enzymes in the regulation of cellular functions: the production of SM, a key phospholipid for the maintenance of lipid raft integrity [9], [10], [11], [12]; the regulation of ceramide, a bioactive lipid often involved in the control of cell proliferation, differentiation, apoptosis and inflammation (for a review see [13], [14], [15], [16], [17]); and the modulation of DAG, a well-established mitogenic lipid also involved in other cellular processes, such as vesicular trafficking [18], [19].

Indeed regulation of SM, ceramide, and DAG has been documented upon modulation of either SMS1 or SMS2 by gene over-expression, by their down-regulation using siRNA or by the use of knock-out animals in the case of SMS2. The role of SMS1 in maintenance of lipid microdomain structure and function *via* modulation of SM levels has been shown in response to Fas ligation, treatment with alkyl-lysophospholipids and CD3 [10], [11], [20]. Similarly, involvement of SMS2 has been demonstrated by using macrophages from SMS2 KO mice treated with lypopolysaccharide or in HEK 293 or THP-1 derived macrophages upon siRNA-mediated down-regulation and treatment with tumor necrosis factor α (TNF-α) [21]. On the other hand, over-expression of SMS1 or SMS2 in Chinese hamster ovary (CHO) cells promoted the formation of detergent-insoluble microdomains and apoptosis induced by TNF [21]. Changes in ceramide levels due to modulation of SMSs might be differently regulated depending on the specific cellular context. In fact, in resting Jurkat and CHO cells over-expression of SMS1 or SMS2 caused an increase of ceramide as part of a general stimulation of sphingolipid synthesis [22] whereas, in Jurkat cells, it prevented accumulation of ceramide in response to photodamage [22]. On the other hand, in HeLa, Jurkat, and Huh cells, siRNA-mediated down regulation of *SMS1* or *SMS2* enhanced accumulation of ceramide [8], [23], [24], [25].

Regulation of DAG by SMSs has been quite elusive [8], [21], [24], [25]. The best characterized cell model for such regulation is represented by HeLa cells, upon stimulation of SMSs activity at the Golgi. Using this model, we previously demonstrated that DAG is produced in this organelle by both SMS1 and SMS2, and we preliminarily proposed the DAG-binding protein, PKD as binding partner of this specific pool of DAG [8].

PKD constitutes a family of serine/threonine-specific kinases that in mammalian cells is composed of three closely related isoforms, PKD1/PKCμ [26], PKD2 [27]and PKD3/PKCν [28]. Activation of PKD at the Golgi occurs after translocation of the kinase to this organelle where its C1 domain binds to DAG [29], [30], [31], [32]. Activated PKD at the Golgi has been shown to promote vesicular fission from the trans golgi network (TGN) [33]. Thus far, formation of DAG at the Golgi is thought to be a consequence of the activation of phospholipase C β3 by the heterotrimeric G protein βγ subunits (Gβγ) [34]. In addition to DAG, recruitment of PKD at the Golgi may also be facilitated by binding of Gβγ to the pleckstrin homology (PH) domain [35] of PKD. The production of DAG at the Golgi also favors the translocation of PKCη, which in turn is activated by Gβγ. Activated PKCη at the Golgi phosphorylates the kinase activation loop domain of PKD at Serines 744–748 and relieves the inhibition of PKD by its own PH domain [36]. Once activated in the Golgi complex, PKD phosphorylates PI4KIIIβ and the ceramide transfer protein (CERT). PI4KIIIβ promotes formation of PI(4)P [37] which binds to CERT *via* its PH domain, recruiting CERT to the Golgi apparatus. CERT facilitates transport of ceramide from the ER to the Golgi for SM synthesis by SMS [38], and phosphorylation of CERT by PKD at the Golgi diminishes CERT's affinity for PI(4)P [39]. Based on these observations, phosphorylation of CERT by PKD is thought to exert a negative feedback on SMS-dependent PKD activation, even though no direct evidence for a role of SMS-derived DAG on the function of endogenous PKD is available in the literature. Finally, active PKD also phosphorylates oxysterol-binding protein (OSBP) which is also recruited to the Golgi *via* PI(4)P. Phosphorylation of OSBP by PKD was found to impair the Golgi localization of OSBP in response to 25-hydroxycholesterol. This in turn affected CERT localization at the Golgi and provided an additional level of regulation to this tightly controlled multistep process [40].

Thus, in order to investigate whether SMS1 and SMS2 regulate TGN functions in a fashion that is compatible with the potential role of PKD as down-stream target of SMSs, we studied the role of SMSs in the vesicular trafficking from the Golgi to the plasma membrane. Here we report that inhibition of SMS1 or SMS2 alters the structure of the Golgi apparatus in a way similar to inhibition of PKD, and that inhibition of SMS1 or SMS2 impairs protein transport from the TGN to the plasma membrane and secretion of an endogenous cargo like insulin. Implications of these findings are discussed.

## Materials and Methods

### Materials

DMEM (Dulbecco's modified Eagle's medium), RPMI 1640 medium, Opti-MEM, trypsin/EDTA solution, FBS (fetal bovine serum) and Penicillin/Streptomycin solution were from Gibco/Invitrogen. Bradford protein assay kit was from BioRad (Cat No: 500-0006). RIA and ELISA kits were from Millipore (Cat. No: RI-13K and EZRMI-13K, respectively).

### Cell culture

Exponentially growing HeLa cells (obtained from the American Type Culture Collection) not exceeding 20 passages were cultured in high glucose DMEM. The medium was supplemented with 10%FBS, 1% Penicillin/streptomycin solution and cells were grown at 37°C in a humidified 5% CO_2_ incubator. The rat INS-1 (823/13) cells were a generous gift from Drs. Buse and Lemasters (Medical University of South Carolina, SC, USA) and Dr. Newgard (Gifford Laboratories for Diabetes Research, University of Texas Southwestern Medical center, Dallas, TX, USA). These cells were cultured in RPMI-1640 medium containing 11.1 mM glucose, 10%FBS, 1%Penicillin/streptomycin solution, 10 mM HEPES, 2 mM L-glutamine, 1 mM sodium-pyruvate and 0.05 mM 2-mercaptoetanol at 37°C in a humidified 5% CO_2_ incubator, and they were used between 76 and 92 population doublings (PDs).

### Down regulation of *SMS1* and *SMS2*

Down regulation of human *SMS1* or *SMS2* in Hela cells was achieved with siRNA oligonucleotides targeting *SMS1*: CTACACTCCCAGTACCTGG (SMS1.3 siRNA) and CACACTATGGCCAATCAGCAA (SMS1.4 siRNA), or *SMS2*: AACCCAAGAGCTTATCCAGTG (SMS2.2 siRNA), and ACCGTCATGATCACAGTTGTA (SMS2.3 siRNA) synthesized by Qiagen, and by using Oligofectamine™ transfection reagent (Invitrogen). The non-specific All Star siRNA sequence from Qiagen (SCR; scrambled siRNA) was used as control. HeLa cells were plated in 35 mm diameter confocal dishes at a density of 4×10^4^ per plate. After 24 hours, cells were transfected with siRNA according to the manufacturer's instructions in a total volume of 1 ml of transfection mixture containing siRNA and Oligofectamine™ in Optimem medium (148 ng of SiRNA). After 6 hours of incubation, 1 ml of DMEM containing 20% FBS was added to the plates. In Hela cells, *SMS1* down-regulation with either siRNA sequence results in more than 70% decrease of mRNA expression and 50–60% reduction of total SMS activity whereas *SMS2* down-regulation with either siRNA sequence results in more than 70% decrease of mRNA expression and 30–40% reduction of total SMS activity. Neither one of the siRNA sequences for one SMS affects the expression of the other isoform [8], [25] (and data not shown).

Down-regulation of rat *SMS1* or *SMS2* in INS-1 (832/13) was achieved using Stealth siRNA oligonucleotide targeting *SMS1*: GGGCAAGACTTTGCTGGCCTTTCTT (Rat SMS1.1 siRNA) and CCTGTACCGGTGTATTACAATGTAT (Rat SMS1.2 siRNA), or *SMS2*: TGGCTCTTTCTGAGATACAAGTCAA (Rat SMS2.1 siRNA) and CAGTTGTGCTGA CACTTACTTACTT (Rat SMS2.2 siRNA) synthesized by Invitrogen, and by using Lipofectamine 2000 transfection reagent (Invitrogen). A non-specific negative control siRNA sequence from Invitrogen (SCR siRNA; scrambled siRNA) was used as control. Cells were either treated with a concentration of 50 nM of each individual siRNA sequence or with a combination of 50 nM of each siRNA sequence targeting the same gene. In the latter case, 100 nM SCR siRNA was used as control (SCR siRNA mix). These siRNA concentrations were found to exert the strongest down-regulation of rat *SMS1* and *SMS2*. Rat INS-1 (832/13) cells were plated in 12-wells plates at a density of 3×10^5^ cells/well. After 24 hours, cells were transfected with siRNA according to the manufacturer's instructions. After 27 hrs from transfection, medium was replaced with 1 ml of fresh complete medium (RPMI medium containing 5 mM glucose, 10% FBS and 1% Penicilin/streptomycin solution, 10 mM HEPES, 2 mM L-glutamine, 1 mM sodium-pyruvate and 0.05 mM 2-mercaptoethanol). After 18 hours, cells were washed in 1 ml HBSS [114 mM NaCl, 4.7 mM KCl, 1.2 mM KH_2_PO_4_, 1.16 mM MgSO_4_, 20 mM HEPES, 2.5 mM CaCl_2_, 25.5 mM NaHCO_3_ and bovine serum albumin (essentially fatty acid free), pH 7.2] containing 3 mM glucose followed by 2 hours incubation in 2 ml of the same buffer (47 hours from transfection). Cells were then collected and frozen at −80°C for RT-PCR or treated for measurements of insulin secretion as described below.

### Real-time (RT)-PCR

Total RNA from Hela or INS-1 cells was extracted using an RNeasy Mini Kit (Qiagen) following the manufacturer's suggested protocol (including the optional DNase and wash steps). The general protocol for RT-PCR for *SMS1* and *SMS2* was performed as indicated by Villani *et al* [8]. The results were normalized to an internal control gene, beta actin. The RT-PCR results were analyzed using Q-Gene® software, which expresses data as the means of normalized expression. The primers were designed using PerkinElmer Primer Express® software. Primer sequences for human SMS1 and SMS2 are described in Villani *et al* [8] whereas primer sequences for rat SMS1 and SMS2 are as follows: *beta Actin*, forward: 5′-GCTACAGCTTCACCACCACA-3′, reverse: 5′-TCTCCAGGGAGGAAGAGGAT-3′; *SMS1*, forward: 5′-TCACAGGCCAGGACCTAATC-3′, reverse: 5′-TGCTCCATCTTCAGGGTCTC-3′; *SMS2*, forward: 5′-AGCCCCACTGAAGCTGTAGA-3′, reverse: 5′-CCACTCTAGGGGAAGCTTGTT-3′.

### Immunofluorescence and confocal microscopy

HeLa cells were plated in 35 mm diameter confocal dishes at a density of 4×10^4^ cells/plate (in 2 ml of 10% FBS containing DMEM medium). After 48 hours, medium was replaced with 1 ml of fresh DMEM containing 10% FBS. In case of treatment with pharmacological inhibitors, 10 to 50 µg/ml of the SMS inhibitor D609 (Enzo Life sciences, Cat. No: ST-330) or 10 to 40 µM of the protein kinase D (PKD) inhibitor CID755673 (TOCRIS Biosciences, Cat.No:3327) were added to the plates and incubated for 4 hours. For down-regulation of SMSs, cells were plated at a density of 3×10^4^ per plate in 35 mm diameter confocal dishes. After 24 hours, cells were transfected as described above with 148 ng SCR, SMS1.4 or SMS2.3 siRNA for 48 hours. Treated cells (both after pharmacological treatment or siRNA-mediated down-regulation) were fixed with 3.7% formaldehyde for 15 min at room temperature (24°C). After washing the plates with PBS, cells were permeabilized with 100% methanol (−20°C) for 5 min. Cells were washed with 1.5% FBS in PBS for 5 min and then blocked with 2.5% FBS in PBS for 1 hour at room temperature. Incubation with the primary antibody was performed using 1∶50 dilution of rabbit anti-human TGN 46 antibodies (from Novus Biological, Cat. No: NB110-40769) or Polyclonal rabbit antibodies against GPP130 (Covance, Cat No: PRB-144C), in 1.5% FBS in PBS containing 0.5% saponin for 3 hours at room temperature or overnight at 4°C. Cells were then washed three times with 1.5% FBS/PBS for 5 min each and incubated with anti-rabbit Alexa Fluor® 488 (1∶400 dilution in PBS containing 1.5% FBS and 0.5% Saponin) for 1 hour at room temperature, in the dark. Cells were washed three times with 1.5% FBS/PBS for 5 min each before analysis at the confocal microscope. Confocal images were captured and processed using an LSM 510 META laser-scanning microscope (Zeiss, Jena Germany).

INS-1 cells were plated in 35 mm confocal dishes at a density of 1×10^5^ cells/plate and were grown to 100% confluence. The standard tissue culture medium containing 11.1 mM glucose was then replaced by 1 ml of fresh medium containing 5 mM glucose. After 14 hours, cells were treated for 4 hours with the SMS inhibitor, D609 (10 to 50 µg/ml), or the PKD inhibitor CID755673 (10 to 40 µM) while control cells received sterile water or DMSO (0.05%), respectively. Cells were fixed and processed for confocal microscopy as described for HeLa cells. Cells were co-stained with rabbit polyclonal anti-GPP130 (Covance) and purified mouse monoclonal anti-rat TGN38 (from BD Transduction Laboratories, Cat No: 610898) (1∶50 dilution). Plates were processed as indicated above, with the exception that anti-rabbit Alexa Fluor® 488 and anti-mouse Alexa Fluor®555 (1∶400 dilution in PBS containing 1.5% FBS and 0.5% Saponin) were used. Series of scans along the Z axis were taken by confocal microscopy and analyzed by velocity 3 software (Improvision).

### VSVG-GFP transport assay

A temperature-sensitive mutant of vesicular stomatitis virus G protein tagged with GFP (VSVG-GFP), kindly provided by Dr. Kai Simons [European Molecular Biology Laboratory (EMBL), Meyerhofstrasse 1, 69117 Heidelberg, Germany], was used to study plasma membrane transport by following the procedure described by Toomre *et al.*, [41] with slight modifications. HeLa cells were plated in 35 mm diameter confocal dishes at a density of 5×10^4^ cells/plate (2 ml of DMEM containing 10% FBS). After 48 hours, cells were transfected with 2 µg of VSVG-GFP plasmid using Lipofectamine 2000 (Invitrogen, Cat no: 11668-019) and incubated at 37°C overnight. Then medium was replaced with fresh DMEM containing 10% FBS and 0.1 M HEPES (pH 7.4) and the plates were shifted to 40°C (temperature at which VSVG-GFP reversibly misfolds and stays in the ER) for five and half hours (**Figure S1**). After incubation, the medium was replaced by DMEM containing 10% FBS, 0.1 M HEPES pH 7.4 and 100 µg/ml of cyclohexamide (to stop newly synthesized protein) and plates were shifted to 32°C (temperature at which VSVG-GFP folds correctly and exits the ER en-route to the Golgi apparatus [42]). After 30 min at 32°C, VSVG-GFP reached the Golgi complex (**Figure S1**) and within 60 min it slowly reached the plasma membrane (**Figure S1**). Beyond 60 minutes, (65 and 70 min) endocytosis of VSVG-GFP was observed in agreement with previous studies [41]. Thus for the studies of the role of SMSs on the trafficking of VSV-GFP to the plasma membrane, 60 min incubation at 32°C was selected. For down-regulation of SMSs, cells were plated at a density of 3×10^4^ per plate in 35 mm diameter confocal dishes. After 24 hours, cells were treated with 148 ng of SCR, SMS1.3, SMS1.4 or SMS2.2, SMS2.3 siRNA or Oligofectamine™ alone (control). After 48 hours of down-regulation, cells were transfected with 2 µg of VSVG-GFP by using Lipofectamine 2000 and they were incubated overnight at 37°C and processed as indicated above. After the five and half hours incubation at 40°C and 1 hour at 32°C, cells were fixed with 3.7% formaldehyde for 15 min at room temperature (24°C). After washing the plates with PBS, the cells were analyzed by confocal microscopy.

For specific detection of plasma membrane VSVG-GFP, monoclonal 8G5F11 antibodies that recognize the extracellular domain of VSVG (kindly provided by Dr. Douglas S. Lyles, Wake Forest University Baptist Medical Center, NC) were used in fixed (non-permeabilized) cells [43]. After fixation, cells were washed once with 1.5% FBS in PBS for 5 min as described above, and incubation with the 8G5F11 primary antibodies was performed in 1.5% FBS in PBS at 1∶50 dilution for 3 hours at room temperature or overnight at 4°C. Cells were washed three times with 1.5% FBS in PBS for 5 min each and incubated with anti-mouse Alexa Flour® 555 conjugated antibodies (1∶400 in 1.5% FBS in PBS) for 1 hour at room temperature in the dark. Cells were washed three additional times with 1.5% FBS/PBS for 5 min each before analysis at the LSM 510 META laser-scanning microscope (Zeiss, Jena Germany).

### Insulin secretion

For insulin secretion assays the protocol by Hohmeier *et al*., [44] with slight modifications was employed. For pharmacological inhibition of SMSs and PKD, rat INS-1 (832/13) cells were plated onto 24-wells plates at a density of 3×10^5^ cells/well and were grown to 100% confluence before assay. The standard tissue culture medium containing 11.1 mM glucose was then replaced by 1 ml of fresh medium containing 5 mM glucose. After 14 hours cells were treated for 4 hours with the SMS inhibitor, D609 (30 µg/ml and 50 µg/ml), or the PKD inhibitor CID755673 (20 µM and 40 µM) while control cells received sterile water or DMSO (0.05%), respectively. Cells were washed in 1 ml HBSS (114 mM NaCl, 4.7 mM KCl, 1.2 mM KH_2_PO_4_, 1.16 mM MgSO_4_, 20 mM HEPES, 2.5 mM CaCl_2_, 25.5 mM NaHCO_3_ and bovine serum albumin [essentially fatty acid free], pH 7.2) containing 3 mM glucose followed by a 2 hours pre-incubation in 2 ml of the same buffer. For siRNA-mediated down-regulation of *SMS1* or *SMS2*, cells were plated and treated as described for INS-1 cells in the paragraph “down-regulation of *SMS1* or *SMS2*”. After the 2 hours incubation in HBSS medium containing 3 mM glucose, the buffer was replaced with 0.8 ml of HBSS containing different concentrations of glucose (3 mM or 8 mM). After 2 hours of static incubation, the medium was collected and spun at 400× *g*, 5 min at 4°C. Supernatant was then used for measuring secreted insulin. Cells were then collected by gently dislodging them from the plates using a cell lifter in the presence of ice-cold HBSS (600 µl) without glucose. Detached cells were combined with pelleted cells from medium, and spun down at 400× *g*, 5 min at 4°C. Pellets were sonicated in 1 M acetic acid containing 0.1% bovine serum albumin and aliquots were used to determine cellular insulin. Insulin measurements from experiments with pharmacological inhibitors were performed by radioimmunoassay (RIA) whereas insulin from siRNA-mediated down-regulation experiments was determined by ELISA. Both RIA and ELISA measurements were performed using rat insulin kits from Millipore and the procedure followed the manufacturer's guide.

In both cases, total insulin content was calculated as cellular plus secreted insulin, and secreted insulin was calculated as percent versus total. Since the percent of secretion in basal conditions (3 mM glucose) was variable, final representation of glucose-stimulated secreted insulin is given as fold change relative to insulin secreted in basal conditions (3 mM glucose).

### Statistical analysis

Statistical analysis of the data was performed using student's two tailed *t*-test and *P*<0.05 was considered statistically significant.

## Results and Discussion

### Modulation of SMS affects the organization of the Golgi in HeLa cells

**Figure 1:**
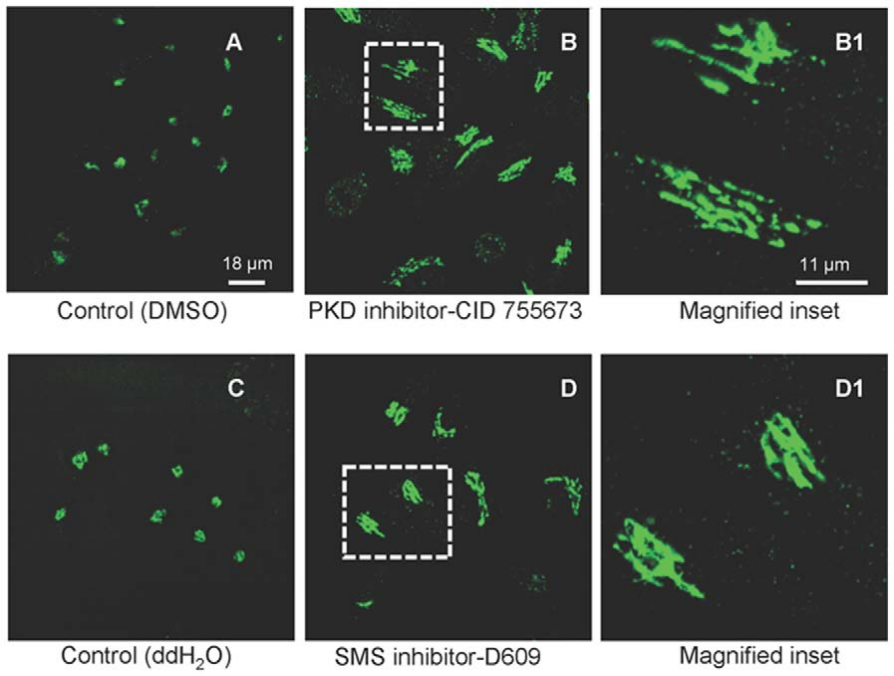
Pharmacological inhibition of SMS causes extensive tubulation of the TGN. HeLa cells were treated with DMSO (control for PKD inhibitor) (1A) or water (control for SMS inhibitor) (1C), and 20 µM of PKD inhibitor (1B) or 30 µg/ml of SMS inhibitor D609 (1D). After 4 hours of incubation, cells were fixed and processed for indirect Immunofluorescence with anti TGN 46 (trans golgi marker) rabbit polyclonal antibodies and stained with anti rabbit Alexa Fluor® 488-conjugated secondary antibodies. Confocal images were captured and processed using an LSM 510 META. Images are representatives of at least three independent experiments. Dashed boxes outline the areas that were magnified.

**Figure 2:**
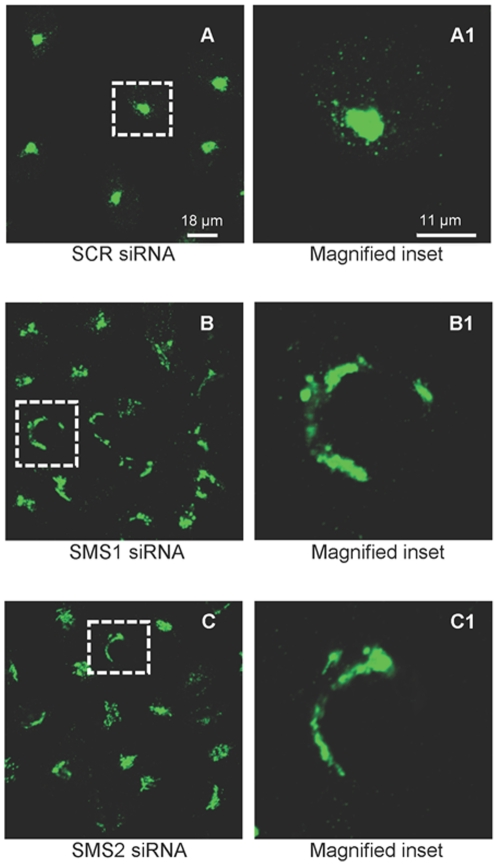
SMS1 or SMS2 inhibition modulates the TGN organization. HeLa cells were transfected using Oligofectamine™ with 148 ng of scrambled siRNA (SCR, 2A), siRNA targeting SMS1 (SMS1.4 siRNA, 2B) or SMS2 (SMS2.3 siRNA, 2C). After 48 hours, cells were fixed and processed for indirect Immunofluorescence with anti TGN 46 (Trans Golgi marker) rabbit polyclonal antibodies and stained with anti rabbit Alexa Fluor® 488-conjugated secondary antibodies (green). Confocal images were captured and processed using an LSM 510 META. Images are representatives of at least three independent experiments. Dashed boxes outline the areas that were magnified.

Since we previously provided direct evidence that both SMS1 and SMS2 possessed the ability of regulating production of DAG at the Golgi and that this pool of DAG is biologically active for recruitment of over-expressed PKD from the cytosol to the Golgi, we hypothesized that PKD might be a downstream target for SMS1 and SMS2 at the Golgi. If this is true, then inhibition of SMSs should induce morphological changes of the Golgi structure similar to those evoked by inhibition of PKD activity. Since PKD activity at the Golgi is required for fission of secretory vesicles from the TGN, inhibition of PKD has been shown to induce tubulation of the TGN [35], [45]. In agreement with these published observations, incubation of HeLa cells with the PKD inhibitor CID755673 (20 µM) induced tubulation (in ∼70% of the cells) of the TGN assessed by staining with the TGN46 marker (**Figure 1** **, panels B and B1**) as compared to the intact organization of the TGN in control cells (**Figure 1A**). At a lower concentration (10 µM) tubulation in ∼40% of the cells was observed (data not shown). Similarly to inhibition of PKD, pharmacological inhibition of SMS activity by D609 (20 µg/ml and 30 µg/ml) also induced significant tubulation of the TGN (∼40% and 60–70% of the cells, respectively) (**Figures 1** **, panels D and D1** for 30 µg/ml D609). To ensure the specificity of the results and to determine if both SMSs are involved in maintenance of the Golgi morphology (since D609 inhibits both SMS1 and SMS2, [24], [46], [47]), the effect of siRNA-mediated down-regulation of either SMS on TGN structure was determined (**Figure 2**). Validated siRNA sequences were used [8], [25], along with a siRNA scrambled control (AllStar, Quiagen). Cells treated with either SMS1 (**Figure 2B**) or SMS2 (**Figure 2C**) siRNA showed tubular protrusions from the TGN reminiscent of inhibited vesicle fission observed upon pharmacological inhibition of either PKD or SMS.

**Figure 3:**
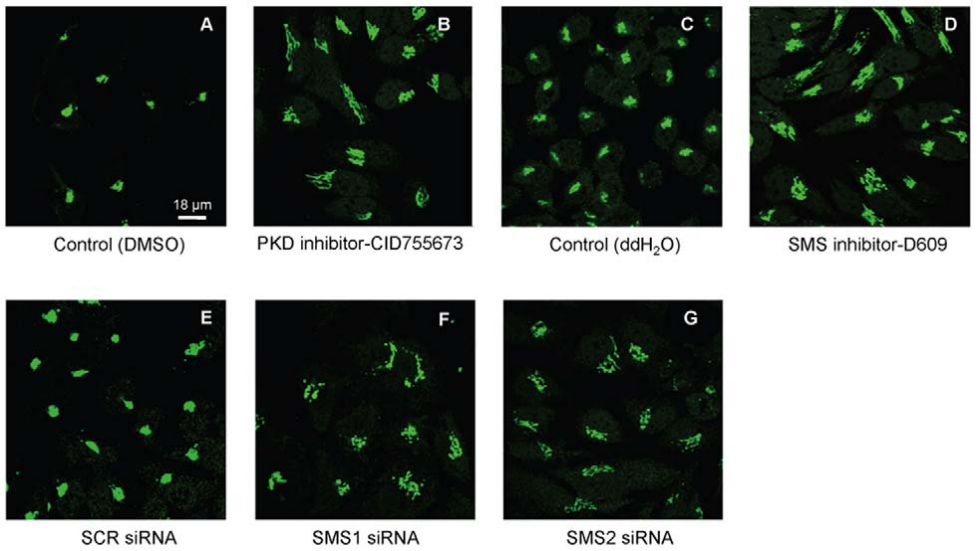
SMS inhibition affects cis Golgi. HeLa cells were treated with DMSO (control for PKD inhibitor) (1A) or water (control for SMS inhibitor) (1C), and 20 µM of PKD inhibitor (1B) or 30 µg/ml of SMS inhibitor D609 (1D). Alternatively, HeLa cells were transfected using Oligofectamine™ with 148 ng of scrambled siRNA (SCR, 2E), siRNA targeting SMS1 (SMS1.4 siRNA, 2F) or SMS2 (SMS2.3 siRNA, 2G). After 4 hours of pharmacological inhibition or 48 hours of siRNA treatment, cells were fixed and were processed for indirect immunofluorescence with anti GPP130 (cis Golgi marker) rabbit polyclonal antibodies and stained with anti rabbit Alexa Fluor® 488-conjugated secondary antibodies (green). Confocal images were captured and processed using LSM 510 META. Images are representatives of at least three independent experiments.

Inhibition of PKD has been reported not only to impair the structure of the TGN but also of the *cis*-Golgi [48]. Thus the effects of pharmacologic and siRNA-mediated inhibition of SMSs on the *cis* compartment of the Golgi stack were assessed by confocal microscopy using GPP130 as marker. In agreement with the literature, inhibition of PKD disrupted the normal morphology of the *cis* compartment promoting tubulation as also observed in the case of the TGN (**Figure 3** **, panel B versus A**). Importantly, inhibition of SMSs (either by D609 or by specific siRNA) exerted a similar effect (**Figure 3** **, panel D versus C** and **panels F or G versus E**). The morphological changes of either *cis*- or *trans-*Golgi were more prominent in cells treated with D609 than in cells in which individual down-regulation of *SMS1* or *SMS2* was induced with siRNA. Since D609 inhibits both SMS1 and SMS2, it is possible that the D609-mediated effect is the result of the additive inhibition of both SMSs as compared to down-regulation of each individual SMS with siRNA.

Collectively, these results indicate that both SMS1 and SMS2 activities are critical to retain the correct morphology of both *cis*-Golgi and TGN, similarly to what was indirectly shown for PKD [48]. The fact that inhibition of either SMS1 or SMS2 affected the morphology not only of the *trans-* but also of the *cis*-Golgi could be the result of the presence of different subpools of SMSs in the different compartments of this organelle. Indeed, in HeLa cells, different reports have localized SMS1 in both *trans*- and *cis*-Golgi [8], [24], [49], and electron microscopy in these cells confirmed that SMS1 is present in both compartments [50]. Importantly, the fact that morphological alteration of both *cis*- and *trans*-Golgi was also appreciated upon inhibition of PKD (**Figures 1B** **and** **3B**) is in agreement with the potential role of PKD as down-stream target for SMSs. The observations that down-regulation of SMSs affects the morphology of the Golgi apparatus seem to disagree with published data showing that down-regulation of *SMS1* did not significantly alter the distribution of alpha-mannosidase II [24]. On the other hand, it is possible that this difference might be due to the fact that in the present study the Golgi morphology was studied using endogenous markers (TGN46 and 38 for *trans*-Golgi and GPP130 for *cis*-Golgi) whereas the published report used an over-expression system which might have masked more subtle changes. Support for a role of SMSs in maintenance of the Golgi structure indirectly comes from experiments involving the vesicle-associated membrane protein-associated proteins, VAPs. In fact, down-regulation of the VAPs, which regulate the binding of CERT to the ER for ceramide loading destined to SM synthesis, caused significant alteration of Golgi morphology [51].

### Effect of SMS1 or SMS2 siRNA on VSVG trafficking

Since PKD is a known regulator of fission of vesicles leaving the TGN en route to the plasma membrane, and we observed that SMSs are also important enzymes regulating Golgi morphology, we wanted to test whether inhibition of SMS would affect protein transport from the TGN to the plasma membrane. To this aim, the effect of siRNA-mediated down-regulation of *SMSs* on trafficking of the VSVG protein tagged with the Green Fluorescent Protein (VSVG-GFP) was studied. A temperature sensitive mutant was employed for these studies. Under the non-permissive temperature of 40°C, the mutant VSVG-GFP reversibly misfolds and is retained in the ER (**Figure S1**) [42]. Thus, in order to synchronize the trafficking of the protein, after transfection and expression of VSVG-GFP at 37°C, the cells were shifted to 40°C for five and half hours (**Figure S1**). Once the trafficking of the mutant VSVG-GFP was synchronized, the cells were shifted to 32°C (temperature at which VSVG-GFP folds correctly and exits the ER en-route to the Golgi apparatus [42]). After 30 minutes at 32°C, VSVG-GFP reached the Golgi complex (**Figure S1**) and within 60 minutes, it reproducibly reached the plasma membrane (**Figure S1**). Beyond 60 minutes, endocytosis of VSVG-GFP was observed in agreement with previous studies [41]. Based on these kinetics, 60 minutes incubation at 32°C was selected for the subsequent studies of the role of SMSs on the trafficking of VSVG-GFP to the plasma membrane.

**Figure 4:**
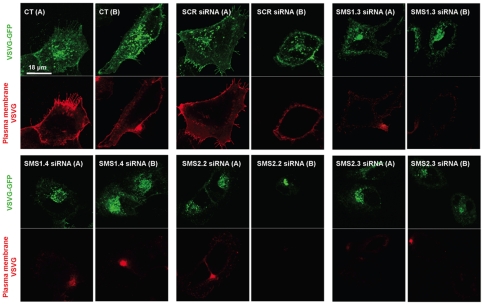
SMS1 and SMS2 regulate protein trafficking from the TGN to the plasma membrane. HeLa cells were treated with Oligofectamine™ transfection reagent alone (CT), 148 ng scrambled control siRNA (SCR siRNA), or siRNA targeting *SMS1* (SMS1.3 siRNA or SMS1.4 siRNA) or *SMS2* (SMS2.2 siRNA or SMS2.3 siRNA) for 48 hours. Cells were then transfected with VSVG-GFP by Lipofectamine 2000. After 16 hours of incubation at 37°C, medium was replaced by DMEM containing 10% FBS and 0.1 M HEPES pH 7.4, and plates were shifted to 40°C for five and half hours. After incubation, the medium was replaced by DMEM containing 10% FBS, 0.1 M HEPES pH 7.4 and 100 µg/ml of cyclohexamide, and the plates were shifted to 32°C for 1 hour. Cells were fixed and were processed for indirect immunofluorescence with anti VSVG mouse monoclonal antibodies (8G5F11) and stained with anti rabbit Alexa Fluor-555-conjugated secondary antibodies. Confocal images for VSVG-GFP (green) and plasma membrane VSVG (red) were captured. Two representative images (A and B) for each experimental sample are presented. Images are representatives of at least three independent experiments.

For down-regulation of *SMSs*, cells were treated with SCR, *SMS1* or *SMS2* siRNA or Oligofectamine™ alone (control) (two different siRNA sequences for each SMS were used) and after 48 hours, cells were transfected with mutant VSVG-GFP and incubated overnight at 37°C. After shifting the cells at 40°C for five and half hours, they were incubated for 60 minutes at 32°C. As shown in **Figure 4** **(CT and SCR siRNA, upper panels)**, cells treated with transfection reagent (no siRNA; CT) or cells treated with scrambled siRNA (SCR siRNA) showed significant localization of VSVG-GFP at the plasma membrane. In order to specifically visualize the VSVG pool present at the plasma membrane, indirect immunofluorescence using an antibody that specifically recognizes an extracellular epitope of VSVG (mAb 8G5F11) was employed. Non-permeabilized CT and SCR siRNA cells were stained with the 8G5F11 antibody and showed a clear plasma membrane localization of VSVG (**Figure 4**, **CT and SCR siRNA, lower panels**). However, when cells were treated with either *SMS1* and to a lesser extent with *SMS2* siRNA, VSVG localization to the plasma membrane after 60 minutes incubation at 32°C was critically impaired (**Figure 4**, **SMS1.3 siRNA, SMS1.4 siRNA, SMS2.2 siRNA, SMS2.3 siRNA**). VSVG-GFP in *SMS1* and *SMS2* down-regulated cells reached the plasma membrane after approximately 80 to 90 minutes at 32°C when control cells had already endocytosed it (data not shown). Thus our results suggest that down-regulation of SMSs significantly slows down (but does not completely block) the trafficking of VSVG-GFP from the Golgi to the plasma membrane, and they demonstrate that both SMS1 and SMS2 activities are important for transport of protein/cargo from the Golgi complex to the plasma membrane.

### Role of SMS on insulin secretion

**Figure 5:**
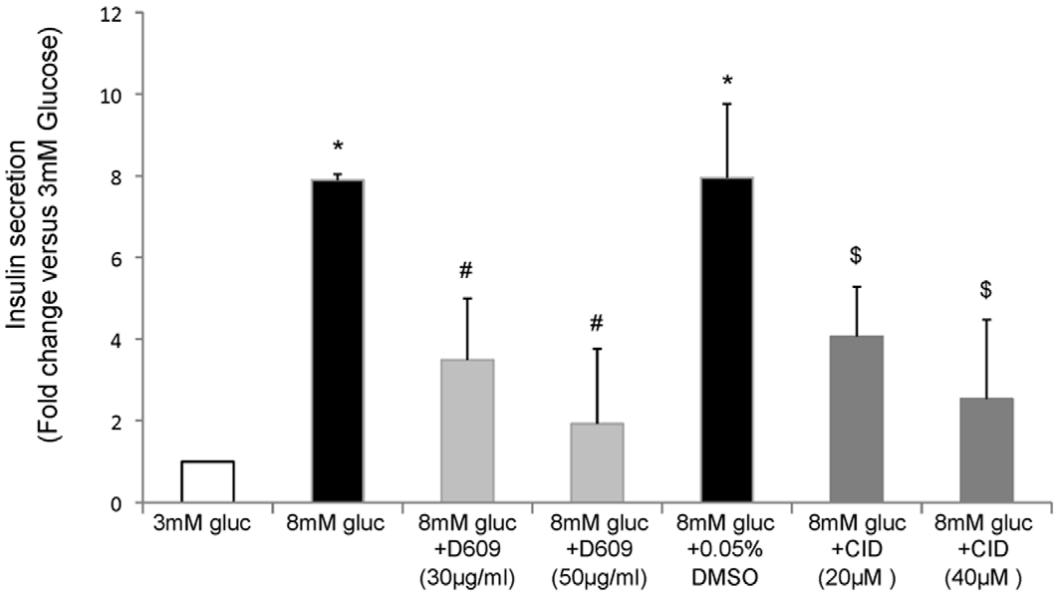
Inhibition of SMS significantly impairs insulin secretion. Insulin secretion in response to 3 and 8 mM glucose in INS-1 derived clone 832/13 was measured by radioimmunoassay. The cells were treated with SMS pharmacological inhibitor, D609 (30 µg/ml and 50 µg/ml) or PKD inhibitor CID755673 (20 µM and 40 µM) at 8 mM glucose for 2 hours. Media and cells were collected separately and insulin levels were determined in each portion. Total insulin content was calculated as cellular plus secreted insulin, and secreted insulin was calculated as percent versus total. Since the percent of secretion in basal conditions (3 mM glucose) was variable, final representation of secreted insulin is given as fold increase relative to insulin secreted in basal conditions (3 mM glucose). The values are representative of at least 4 independent experiments. A *p* value<0.05 was considered significant and is indicated by: * versus 3 mM glucose; # versus 8 mM glucose; and $ versus 8 mM glucose DMSO.

In order to further test the involvement of SMSs in regulation of processes that are controlled by PKD at the TGN, the effect of inhibition of SMSs on secretion of insulin was determined. In fact, it was recently shown by Sumara and colleagues [48], that inhibition of PKD impaired insulin secretion in rat INS-1 cells. Thus the effect of pharmacological inhibition of SMSs on glucose-induced insulin secretion by INS-1 was determined. Inhibition of PKD by CID755673 was used as positive control. As shown in **Figure 5**, an increase of insulin secretion was observed when control cells (3 mM glucose) were stimulated with an higher concentration of glucose (8 mM) demonstrating the expected responsiveness of these cells to glucose stimulation. Also an expected dose dependent decrease of glucose-stimulated insulin secretion was observed when PKD was inhibited (20 µM and 40 µM of CID755673), confirming the published involvement of PKD in this process. Importantly, inhibition of SMS was also found to significantly dose dependently repress insulin secretion in response to glucose stimulation, demonstrating the ability of SMS activity to regulate secretion of endogenous cargo.

**Figure 6:**
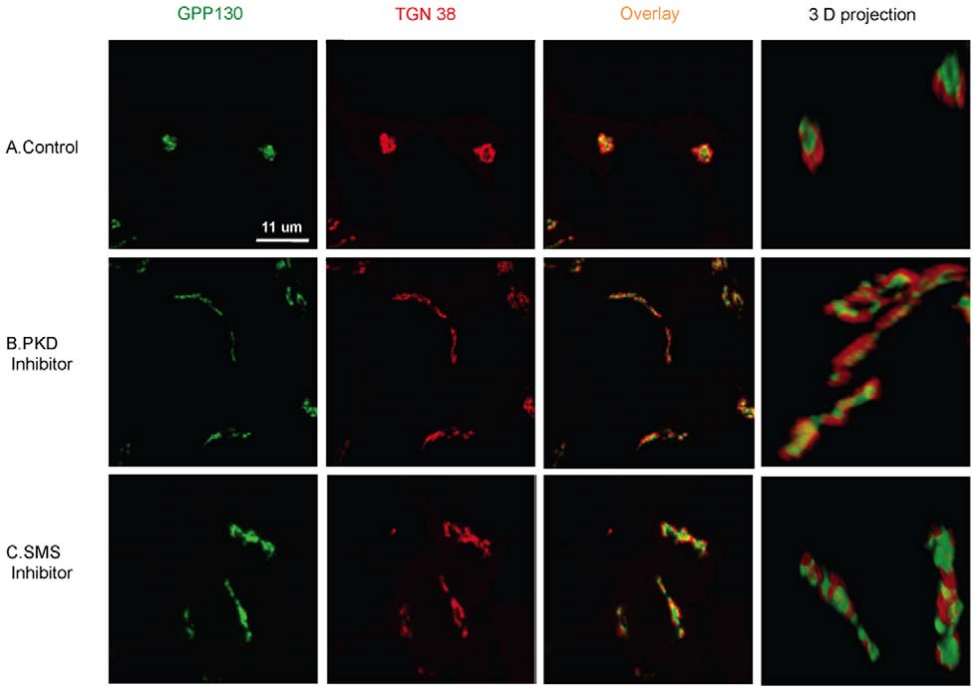
Pharmacological inhibition of SMS causes extensive tubulation of cis Golgi and TGN in INS-1 cells. INS-1 cells were treated with DMSO and water (control), and 20 µM of PKD inhibitor or 30 µg/ml of SMS inhibitor D609. After 4 hours of incubation, cells were fixed and processed for indirect Immunofluorescence with rat anti TGN 38 (TGN marker) mouse monoclonal antibodies and anti mouse Alexa Fluor®555-conjugated secondary antibodies (red), and anti GPP130 (cis Golgi marker) rabbit polyclonal antibodies and anti rabbit Alexa Fluor® 488-conjugated secondary antibodies (green). Confocal images were captured and processed using LSM 510 META. Colocalization in 3D images was achieved using velocity 3 software (improvision). Images are representatives of at least two independent experiments.

In order to make sure that the mechanism of regulation of insulin secretion by PKD and SMS was the same in INS-1 as in HeLa cells, the effect of inhibition of PKD and SMS on the morphology of the Golgi complex in INS-1 cells was determined. For this, mouse monoclonal antibodies against rat TGN38 and rabbit polyclonal antibodies against rat GPP130 were employed as TGN and *cis*-Golgi markers, respectively (**Figure 6**). INS-1 cells were co-stained after treatment with the PKD inhibitor, CID755673 (20 µM) or the SMSs inhibitor, D609 (30 µg/ml). Similarly to HeLa cells, INS-1 cells treated with either the PKD or SMS inhibitor showed tubular structure of the TGN and *cis*-Golgi, which was best appreciated using the velocity 3 (Improvision) software for three-dimensional representation.

**Figure 7:**
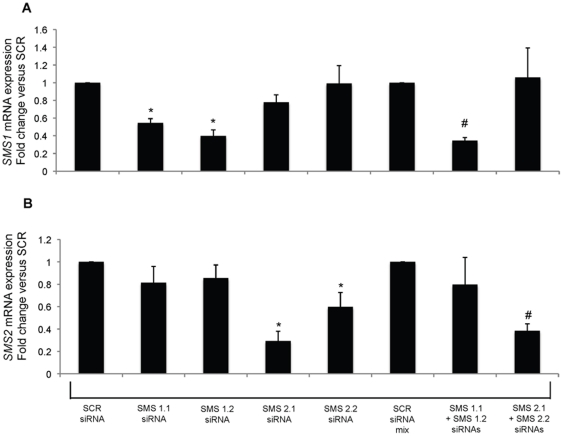
Down-regulation of *SMS1* and *SMS2* in Rat INS-1 (832/13) cells. SMSs were downregulated by specific siRNAs. Rat INS-1 cells were either treated with a concentration of 50 nM of each individual siRNA sequence or with a combination of 50 nM of each siRNA sequence targeting *SMS1* (SMS1.1+SMS1.2 siRNAs) or *SMS2* (SMS2.1+SMS2.2 siRNAs) for 47 hours. In the latter case, 100 nM SCR siRNA was used as control (SCR siRNA mix). Cells were collected at 47 hours, total RNA was extracted, and RT-PCR was performed using specific primers for *SMS1* or *SMS2* and *β-actin*. The RT-PCR results were analyzed using Q-Gene® software, and expressed as fold change versus the respective SCR controls. Panel (A) represents expression of *SMS1* and panel (B) represents expression of *SMS2*. The values are representative of at least 3 independent experiments. A *p* value<0.05 was considered significant and is indicated by: * versus SCR siRNA and # versus SCR siRNA mix.

**Figure 8:**
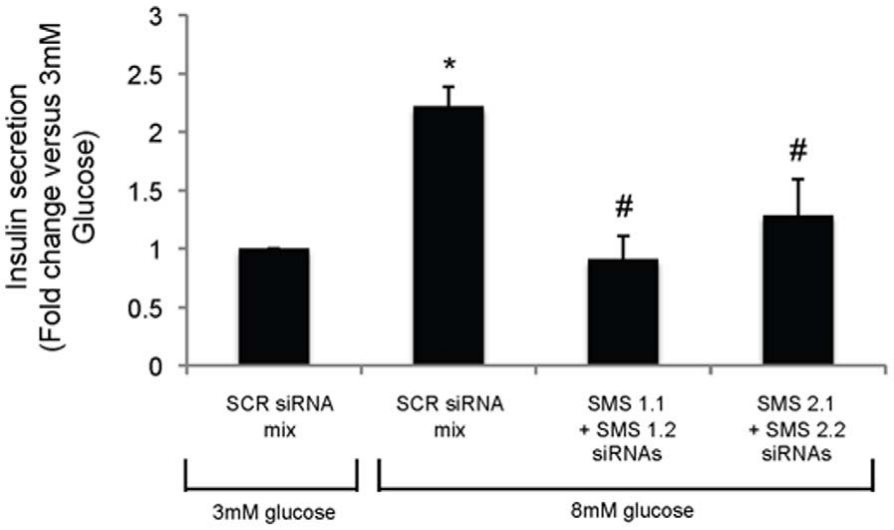
Down-regulation of *SMS1* or *SMS2* significantly reduces insulin secretion in Rat INS-1 (832/13) cells. Insulin secretion in response to 3 and 8 mM glucose in INS-1 derived clone 832/13 was measured by ELISA. Cells transfected with SCR siRNA mix were incubated in the presence of 3 mM or 8 mM glucose whereas cells transfected with *SMS1* siRNAs mix or *SMS2* siRNAs mix were incubated in the presence of 8 mM glucose for 2 hours. Media and cells were collected separately and insulin levels were determined in each portion. Total insulin content was calculated as cellular plus secreted insulin, and secreted insulin was calculated as percent versus total. Since the percent of secretion in basal conditions (SCR siRNA mix 3 mM glucose) was variable, final representation of secreted insulin is given as fold change versus insulin secreted in basal conditions (3 mM glucose). The values are representative of at least 3 independent experiments. A *p* value<0.05 was considered significant and is indicated by: * versus SCR siRNA mix 3 mM glucose and # versus SCR siRNA mix 8 mM glucose.

In order to rule out off-target effects of D609, the effect of siRNA-mediated down-regulation of *SMS1* and *SMS2* on insulin secretion was also determined. Thus two siRNA targeting sequences for rat *SMS1* or *SMS2* each were tested individually or in combination on their effectiveness in repress gene expression (**Figure 7**). As shown in **Figure 7A**, treatment with a 50∶50 combination of the two siRNA sequences targeting *SMS1* resulted in a more effective down-regulation of this gene compared to each sequence used individually, therefore the combination of two siRNA sequences was used to determine the effect of SMS1 or SMS2 on insulin secretion. Importantly, the siRNA sequences targeting either SMS did not significantly affect the expression of the other SMS. As shown in **Figure 8**, down-regulation of either *SMS* induced a significant reduction of glucose stimulated insulin secretion similar to that observed upon pharmacological inhibition. The less prominent stimulation of insulin secretion induced by 8 mM glucose (a little over 2 fold) in this case as compared to that reported in **Figure 5** (∼7 fold) might be due to the lower sensitivity of the ELISA method (used in this case) as compared to the RIA assay used for **Figure 5**.

To the best of our knowledge, this is the first evidence of the involvement of SMS in insulin secretion potentially through a PKD mediated mechanism. Based on these results, it is possible that one of the phenotypes of SMS1 or SMS2 knock-out mice could be a defect in glucose metabolism due to impaired insulin secretion. This hypothesis is indirectly supported by the observation that in Drosophila, loss of a functional CERT caused increased glucose levels which could be due in part by impaired secretion of drosophila insulin like peptides (DILP) [52], [53]. While this paper was under revision, a manuscript reporting a defect in insulin secretion in *SMS1* KO mice was published [54]. This is an exciting observation that strengthens our results by confirming the involvement of SMS1 in this process. Based on our results, we propose that loss of *SMS1* impairs PKD-mediated insulin secretion in these mice, and that impairment of this pathway contributes to their phenotype.

In conclusion, in this study we provide the first direct evidence for a role of both SMS1 and SMS2 as regulators of protein trafficking and secretion from the Golgi apparatus. In this report we identify insulin as one of the protein cargos regulated by SMSs activity. On the other hand, one important standing question is how generalized is the effect exerted by modulation of SMS1 or SMS2 on secretion. Perhaps this could depend upon the level of activity of these proteins in different tissues. Interestingly, in a parallel study, we found that SMS1 and SMS2 regulate extracellular killing of the fungus *Cryptococcus neoformans* by neutrophils [55] by regulating the presence/activity of an antifungal factor in the medium. Since we found that modulation of PKD in the neutrophil model has an effect similar to that observed upon modulation of SMSs, it is possible that SMSs regulate the secretion of microbial antifungal factors in a PKD-dependent manner.

Finally, another important standing question is the existence of more targets for DAG produced at the Golgi by SMS1 and/or SMS2. In fact, in addition to PKD, DAG targets and activates other proteins containing C1 domains such as classical and novel protein kinase Cs (PKCs), Diacylglycerol Kinase, RasGRPs, Chimaerins, and Munc13s [56]. Among these, PKCδ, PKCη and Munc13 have been all described to translocate to the Golgi apparatus making them potential target candidates for Golgi SMSs [18], [36], [45], [57], [58], [59]. Most interestingly, PKCδ has also been recently linked to glucose stimulated insulin secretion [60] and, in the course of preliminary experimentation, we found that stimulation of SMS activity at the Golgi promoted translocation of PKCδ to this organelle, making this particular PKC an additional potential target involved in the regulation of SMS-mediated insulin secretion.

## Supporting Information

Figure S1 **Optimization of a cellular system for protein transport and secretion.** HeLa cells were transfected with VSVG3-GFP using Lipofectamine 2000. After overnight incubation, medium was replaced with DMEM containing 10% FBS and 0.1 M HEPES pH 7.4. Plates were shifted to 40°C and incubated for five and half hours, and some were then fixed (A). After incubation, medium was changed to DMEM containing 10% FBS, 0.1 M HEPES pH 7.4 and 100 µg/ml of cyclohexamide, and the cells were shifted to 32°C for up to 70 minutes. Cells were fixed at 30 min (B), 35 min (C), 40 min (D), 45 min (E), 50 min (F), 55 min (G), 60 min (H), 65 min (I) and 70 min (J) and analyzed by confocal microscopy. Images are representatives of at least three independent experiments.
(TIF)
