# Supplementary figures and images for: Sphingomyelin Synthases Regulate Protein Trafficking and Secretion
Source: PLoS One. 2011 Sep 27;6(9):e23644. doi: 10.1371/journal.pone.0023644 (PMC3181250; doi:10.1371/journal.pone.0023644)

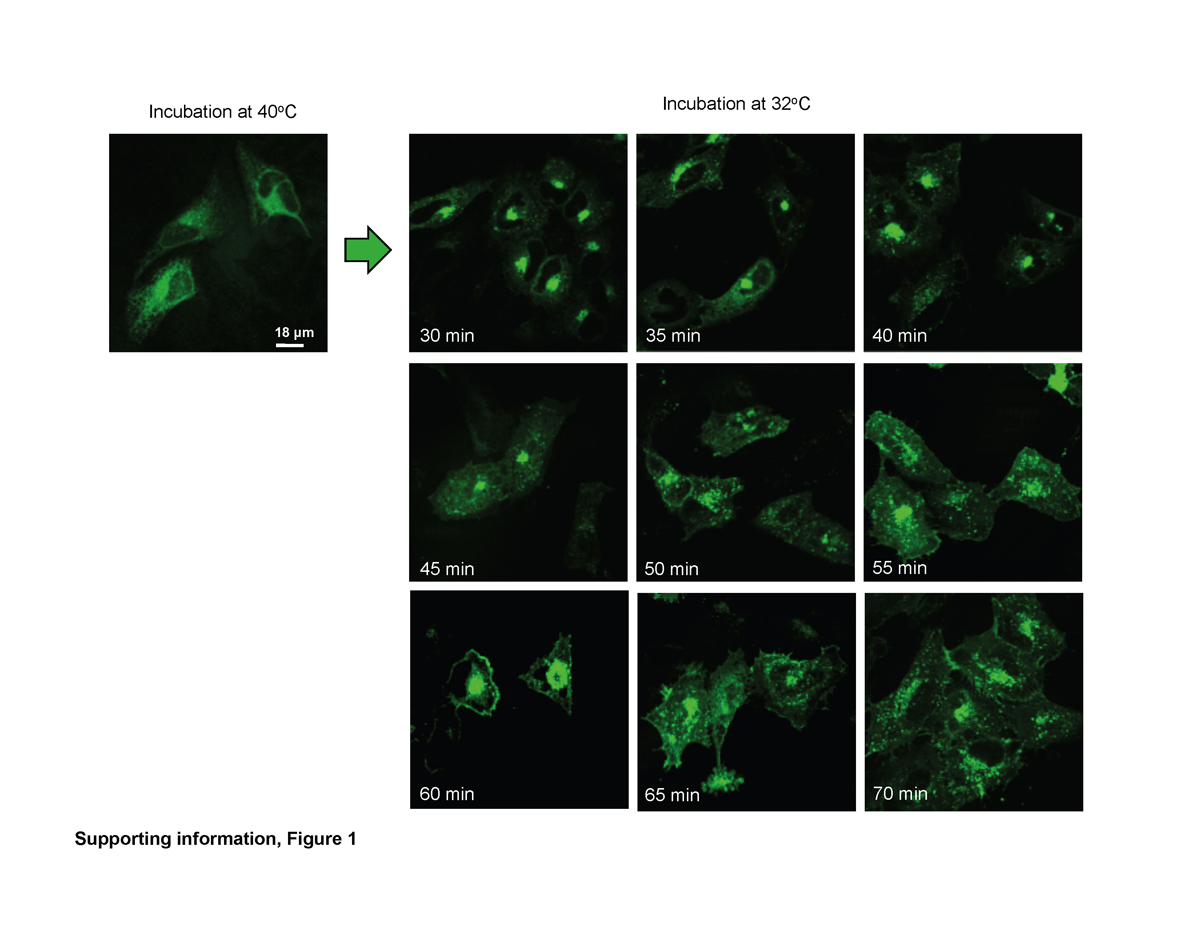

Supplement: Figure S1 — Optimization of a cellular system for protein transport and secretion. HeLa cells were transfected with VSVG3-GFP using Lipofectamine 2000. After overnight incubation, medium was replaced with DMEM containing 10% FBS and 0.1 M HEPES pH 7.4. Plates were shifted to 40°C and incubated for five and half hours, and some were then fixed (A). After incubation, medium was changed to DMEM containing 10% FBS, 0.1 M HEPES pH 7.4 and 100 µg/ml of cyclohexamide, and the cells were shifted to 32°C for up to 70 minutes. Cells were fixed at 30 min (B), 35 min (C), 40 min (D), 45 min (E), 50 min (F), 55 min (G), 60 min (H), 65 min (I) and 70 min (J) and analyzed by confocal microscopy. Images are representatives of at least three independent experiments. (TIF) [file pone.0023644.s001.tif]
